# A Sulfonamide Intermediate Elicits Therapeutic Effect Against Sepsis by Enhancing Neutrophil Maturation

**DOI:** 10.1111/jcmm.71346

**Published:** 2026-09-04

**Authors:** Ji Ye Park, Yu Sun Jeong, Myeongsu Shin, Jiwoo Choi, Jiwon Shin, Leezhi Kwon, Kanghyun Lyu, Hwangi Jo, Youngjoo Byun, JaeHyung Koo, Yoe‐Sik Bae

**Affiliations:** ^1^ Department of Biological Sciences Sungkyunkwan University Suwon Republic of Korea; ^2^ College of Pharmacy Korea University Sejong Republic of Korea; ^3^ Department of New Biology DGIST Daegu Republic of Korea; ^4^ Department of Metabiohealth Sungkyunkwan University Suwon Republic of Korea; ^5^ Interdisciplinary Major Program in Innovative Pharmaceutical Sciences Korea University Sejong Republic of Korea; ^6^ Korea Brain Research Institute (KBRI) Daegu Republic of Korea

**Keywords:** G protein‐coupled receptor, inflammation, neutrophils, sepsis, sulfonamide

## Abstract

Sepsis, a life‐threatening condition characterized by systemic inflammation and immune dysregulation, remains a major cause of mortality worldwide. Here, we identify a synthetic sulfonamide intermediate, 1‐[5‐(2‐fluorophenyl)furan‐2‐yl]‐N‐(4‐methylbenzyl)methanamine (FMM), as a host‐directed therapeutic candidate that enhances neutrophil maturation and antimicrobial function via autophagy. In a *Pseudomonas aeruginosa* ‐induced sepsis model, FMM administration markedly improved survival and attenuated inflammatory cytokine levels while reducing tissue injury and immune cell apoptosis. Furthermore, FMM promoted bone marrow neutrophil maturation, increased reactive oxygen species generation, and enhanced neutrophil extracellular trap formation. FMM also activated GPCR‐mediated G_βγ_–PLC signalling, which triggered intracellular calcium and ERK/p38 phosphorylation. Notably, FMM lacked direct bactericidal activity, suggesting its therapeutic effects are mediated by host immune modulation rather than pathogen targeting. Collectively, these findings demonstrate that FMM exerts potent pharmacological effects on neutrophil function and represents a promising lead compound for developing host‐directed immunomodulatory therapies for sepsis.

## 1 Introduction

Sepsis, a dysregulated host response causing life‐threatening organ dysfunction, remains a major global health challenge [1, 2]. Current treatments, primarily antimicrobial therapy and supportive care [3], still result in poor clinical outcomes [4, 5]. Antibiotics are essential for pathogen control, yet they often prove insufficient because sepsis fundamentally involves a dysregulated host immune response, not merely an uncontrolled infection [6]. Therefore, there is an urgent need to develop novel sepsis treatments that move beyond antibiotics, specifically targeting the intricate immune dysregulation to restore a balanced host response.

Neutrophils, the most prevalent leukocytes in circulation, represent the initial immune defence against infection [7]. These cells undergo a highly regulated maturation process in the bone marrow, transitioning from myeloblasts to mature polymorphonuclear neutrophils through distinct developmental stages characterized by progressive expression of surface markers such as Ly6G and CD101 [8]. Mature neutrophils eliminate microbes mainly through phagocytosis within phagolysosomes [9], degranulation releasing antimicrobial enzymes such as elastase and myeloperoxidase [9], and producing reactive oxygen species (ROS) via NADPH oxidase, which generate toxic molecules to kill pathogens [10]. Additionally, neutrophils can form neutrophil extracellular traps (NETs), web‐like DNA structures that trap pathogens and prevent their dissemination [11]. However, neutrophil function becomes critically dysregulated during sepsis, with impaired phagocytic capacity, defective ROS production, and altered degranulation patterns contributing to inadequate pathogen clearance [12]. Furthermore, sepsis also disrupts bone marrow granulopoiesis, resulting in the premature release of immature neutrophils with diminished antimicrobial function [12], which are associated with adverse clinical outcomes [13]. Therefore, strategies aimed at promoting neutrophil maturation and enhancing their antimicrobial effectiveness represent promising therapeutic approaches for sepsis treatment.

Recent research suggests synthetic intermediates from chemical synthesis processes may exhibit intrinsic biological activities [14]. Synthetic intermediates, previously considered transient steps toward final products, are increasingly recognized as valuable bioactive compounds. For example, β‐lactam intermediates such as 6‐aminopenicillanic acid suppress bacterial growth and stimulate ROS production [15]. Synthetic intermediates of halichlorine containing an azaspiro core structure have been shown to induce apoptosis [16]. Furthermore, fungal‐derived decalin‐containing diterpenoid pyrone intermediates inhibit amyloid‐β aggregation, suggesting potential applications in neurodegenerative diseases such as Alzheimer's disease [17]. These findings suggest that synthetic intermediates represent a novel class of bioactive compounds with therapeutic potential.

Among various compound classes, sulfonamide derivatives are increasingly recognized as promising therapeutic candidates due to their diverse biological activities [18]. Sulfonamides are organic compounds containing the functional group R–SO_2_NH_2_ and are classified into antibiotic and non‐antibiotic sulfonamides. Antibiotic sulfonamides like sulfamethoxazole inhibit bacterial folate synthesis [19]. In contrast, non‐antibiotic sulfonamides exhibit a wide range of pharmacological effects by targeting enzymes and receptors involved in inflammation, cancer, diabetes, and neurological disorders [20, 21, 22]. Sulfasalazine, a representative non‐antibiotic sulfonamide, has been shown to suppress inflammasome activation and inhibit IL‐1β production in peripheral blood mononuclear cells, demonstrating its capacity to modulate innate immune signalling under chronic inflammatory conditions such as HIV‐1 infection [23]. Sulfasalazine promotes NET formation via lipid oxidation mechanisms [24]. In this study, we aimed to examine synthetic intermediates of sulfonamide synthesis for their ability to modulate neutrophil function and to identify bioactive candidates with therapeutic potential in sepsis. We further evaluated the anti‐septic efficacy of the identified compound, *1‐(5‐(2‐fluorophenyl)furan‐2‐yl)‐N‐(4‐methylbenzyl)methanamine* (FMM), in a *Pseudomonas aeruginosa* ( *P. aeruginosa* )‐induced sepsis model and elucidated its mechanism of action, focusing on neutrophil maturation, antimicrobial function, autophagy induction, and GPCR‐mediated signalling.

## 2 Materials and Methods

### 2.1 Synthesis of Sulfonamide Intermediates

The synthesis of FMM is summarized in Figure S1. First, 5‐bromofuran‐2‐carbaldehyde (**1**) was converted into the corresponding aldehyde (**2**) by using the Suzuki coupling reaction. In the second step, compound (2) was transformed into FMM by reductive amination with *p*‐tolylmethanamine. The synthesis of sulfonamide intermediates (KB‐4577, KB‐4578, KB‐4579, KB‐4580, KB‐4582, KB‐4583, KB‐4584) is described in Figure S1.

The synthesis of *5‐(2‐Fluorophenyl)furan‐2‐carbaldehyde* (**2**) was conducted as follows: a mixture of the 5‐bromofuran‐2‐carbaldehyde (2.0 g, 11.4 mmol), Na_2_CO_3_ (2.4 g, 22.9 mmol, 2.0 eq), and tetrakis(triphenylphosphine)palladium (264 mg, 0.229 mmol, 0.02 eq) in 1,2‐dimethoxyethane (20 mL) and water (10 mL) was purged with argon. The reaction mixture was sonicated at room temperature for 5 min, and (2‐fluorophenyl)boronic acid (2.0 g, 14.3 mmol, 1.25 eq) in ethanol (4 mL) was added. The reaction mixture was stirred at 90°C for 90 min. After the reaction mixture was cooled to room temperature, the reaction mixture was diluted with ethyl acetate (EtOAc). The organic layer was washed with aqueous NaHCO_3_ and brine, dried over anhydrous MgSO_4_, and concentrated *in vacuo*. The residue was purified by column chromatography on a silica gel (Hexane:EtOAc = 95:5) to afford compound (2) as a white solid (1.13 g, 52%). *R*_f_ = 0.2 (Hexane:EtOAc = 95:5); ^1^H NMR (600 MHz, CDCl_3_) δ 9.69 (s, 1H), 8.02 (td, *J* = 8.4, 1.7 Hz, 1H), 7.40–7.36 (m, 1H), 7.35 (t, *J =* 3.6 Hz, 2H), 7.26 (td, *J* = 8.4, 0.9 Hz, 1H), 7.17 (ddd, *J* = 11.2, 8.4, 0.9 Hz, 1H), 7.03 (t, *J* = 3.6 Hz, 2H).

To a solution of 5‐(2‐fluorophenyl)furan‐2‐carbaldehyde (**2**) (300 mg, 1.58 mmol) in methanol (5 mL) was added *p*‐tolylmethanamine (200 μL, 1.58 mmol, 1.0 eq). The reaction mixture was stirred at room temperature for 2 h and cooled at 0°C. NaBH_4_ (179 mg, 4.74 mmol, 3.0 eq) was added to the reaction mixture. The mixture was stirred at room temperature until complete conversion was monitored by TLC analysis. The reaction was quenched by saturated aqueous NaHCO_3_ and the excess solvent was removed. The residue was diluted with EtOAc and the organic layer was washed with water and brine, dried over anhydrous MgSO_4_, and concentrated *in vacuo*. The crude was purified by column chromatography on a silica gel (Hexane/EtOAc = 8:2) to afford FMM as a white solid (316 mg, 68%). *R*_f_ = 0.2 (Hexane/EtOAc = 8:2); ^1^H NMR (600 MHz, CDCl_3_) δ 7.82 (td, *J* = 7.8, 2.0 Hz, 1H), 7.24 (d, *J* = 7.8 Hz, 2H), 7.23–7.19 (m, 1H), 7.18 (dd, *J* = 7.8, 2.0 Hz, 1H), 7.15 (d, *J* = 7.8 Hz, 2H), 7.10 (ddd, *J* = 11.1, 7.8, 2.0 Hz, 1H), 6.79 (t, *J* = 3.2 Hz, 1H), 6.32 (d, *J* = 3.2 Hz, 1H), 3.86 (s, 2H), 3.81 (s, 2H), 2.35 (s, 3H); HRMS (ESI) m/z calculated for C_19_H_19_FNO^+^ [M + H]^+^: 296.1445; found: 296.1456. C_11_H_8_FO^+^ [M – C_8_H_10_N]^+^: 175.0554; found: 175.0558.

### 2.2 Animals and an Experimental Sepsis Model

Eight‐week‐old male C57BL/6 mice were purchased from DBL (Eumseong, Korea). All experiments involving animals received the approval of the Institutional Review Committee for Animal Care and Use at Sungkyunkwan University. For the *P. aeruginosa* ‐infection models, *P. aeruginosa* (6 × 10^6^ CFUs per head for sublethal dose, 1.5 × 10^7^ CFUs per head for survival challenge) were intraperitoneally injected. Survival was monitored once daily for 10 days. *P. aeruginosa* cultures were grown for 16 h at 37°C in Luria‐Bertani (LB) broth (Sigma‐Aldrich).

### 2.3 Isolation of Mouse Bone Marrow Neutrophils

Bone marrow cells were isolated from femurs and tibias with sterile PBS. The cell suspension was centrifuged at 1500 rpm for 5 min at 4°C. Next, the resuspended cells were carefully layered onto a discontinuous Percoll gradient consisting of 45% and 60% Percoll (Cytiva, Marlborough, MA, USA), and centrifuged at 2580 rpm for 25 min at 4°C with low brake. Cells were isolated on the 45%–60% interface layer, and RBCs were removed by hypotonic lysis. Isolated cells were confirmed to be > 95% Ly6G‐positive by flow cytometric analysis using a FACS Canto II (BD Biosciences, San Jose, CA, USA).

### 2.4 Measurement of Intracellular Calcium

Intracellular calcium measurement was carried out as previously reported [25]. Briefly, fura‐2 loaded unstimulated neutrophils were stimulated with FMM (50 μM), synthetic intermediates of sulfonamides, or vehicle. The changes in fluorescence ratios (340/380 nm) were monitored using a spectrofluorophotometer (RF5301PC, SHIMADZU, Tokyo, Japan).

### 2.5 Analysis of NET Formation by Fluorescence Microscopy

Isolated unstimulated neutrophils (4 × 10^5^/ml) were seeded on 0.01% poly‐L‐lysine‐coated 24‐well plates. Neutrophils were incubated with *P. aeruginosa*‐derived LPS (50 μg/mL) or ionomycin (5 μM) at 37°C and 5% CO_2_ for 4 h. Stimulated neutrophils were fixed with 4% formaldehyde in PBS for 15 min at room temperature. After washing the samples with RPMI 1640 medium, extracellular DNA was stained with 100 nM SYTOX Orange (Thermo Fisher Scientific) in RPMI 1640 medium for 15 min at room temperature and quantified by fluorescence microscope (Optinity, Korea Lab Tech, Seongnam, Korea).

### 2.6 Measurement of ROS

Isolated unstimulated neutrophils stimulated with FMM (30 μM) were treated with 5 μM DCF‐DA reagent (Thermo Fisher Scientific) in serum‐free RPMI 1640 medium at 37°C for 30 min. The levels of cellular ROS were measured with flow cytometry (FACS Canto II).

### 2.7 Degranulation Assay

Degranulation was measured using a β‐hexosaminidase assay, as previously reported [26]. Briefly, isolated unstimulated neutrophils were incubated with vehicle or FMM (10, 30 μM) for 30 min. Both supernatants and lysates were incubated with the substrate solution at 37°C and 5% CO_2_ for 2 h. O.D. was measured at 405 nm using a spectrophotometer (BioTek EL800).

### 2.8 Measurement of Cytokines

Cytokine levels were measured by enzyme‐linked immunosorbent assay (ELISA) using matched antibody pairs or commercial kits according to the manufacturers' protocols. Samples included peritoneal lavage fluid, serum, and cell‐free supernatants from mouse neutrophils stimulated with *P. aeruginosa* ‐derived LPS (1 μg/mL) for 24 h in the presence or absence of FMM (10, 30 μM). ELISA kits for TNF‐α, IL‐6, IL‐10, and IFN‐γ were obtained from Thermo Fisher Scientific (Waltham, MA, USA), and the assay for IL‐1β was performed using a kit from Mabtech (Nacka Strand, Sweden).

### 2.9 Western Blot Analysis of p65 Translocation and MAPK Phosphorylation

For subcellular fractionation, the Nuclear and Cytoplasmic Extraction kit (Thermo Fisher Scientific) was used. Cytoplasmic and nuclear proteins were sequentially extracted using cytoplasmic and nuclear extraction buffers, respectively. For MAPK signalling analysis, whole‐cell lysates were prepared using RIPA buffer (iNtRON Biotechnology) supplemented with protease inhibitor cocktails (Sigma, St. Louis, MO, USA). Proteins from both fractionation and whole‐cell lysates were separated by SDS‐PAGE and transferred onto nitrocellulose membranes. The membranes were probed with antibodies against p‐p65, IκB, β‐actin, Lamin B1, p‐ERK, t‐ERK, p‐p38, and t‐p38. Antibodies used in Western blot are listed in Table S1.

### 2.10 Tissue Histology and TUNEL Assay

Mice were sacrificed at 24 h after infection. The lungs, liver and spleen were isolated for paraffin processing. Tissues were fixed in neutral buffered formalin, processed and embedded in paraffin, sliced into 4.5 μm sections, and stained with haematoxylin and eosin for microscopic analysis. For terminal deoxynucleotidyl transferase dUTP nick end labelling (TUNEL) assay, the sections were permeabilized with 100 mM sodium citrate at 70°C for 30 min and incubated with normal goat serum at room temperature for 30 min for blocking. After washing, TUNEL staining was performed using an In Situ Cell Death Detection Kit (Sigma‐Aldrich) according to the manufacturer's instructions.

### 2.11 Determination of Colony‐Forming Units (CFUs)

Peritoneal lavage fluid was collected from anaesthetised mice 24 h after infection. The fluid was serially diluted (1:100 and 1:1000), plated on LB agar plates, and incubated at 37°C for 16 h. CFUs were manually counted.

### 2.12 Bacterial Growth Assay

Bacterial suspension (6 × 10^6^ CFUs/ml) was incubated with FMM at concentrations ranging from 3.125 to 100 μM in LB broth at 37°C for 16 h. Bacterial culture without the test compound was used as the untreated control. After 16 h, bacterial growth was measured at O.D. 550 nm using a microplate reader (BioTek EL800).

### 2.13 Antibodies and Flow Cytometry Analysis

Antibodies to Ly6G (1A8), Siglec‐F (E50‐2440), CD45 (30‐F11), MHCII (M5/114.15), CD11b (M1/70), NK1.1 (PK136), CD4 (RM4‐5), CD3e (145‐2C11), CD19 (1D3) and CD101 (Moushi101) were purchased from Thermo Fisher Scientific (Waltham, MA, USA). Antibodies to Ly6C (HK1.4), F4/80 (BM8), c‐kit (2B8), CD11c (N418) and CD8a (53–6.7) were purchased from BioLegend (San Diego, CA, USA). Data were analysed using Cytek Aurora and Flowjo analytical software (BD Biosciences, San Jose, CA, USA). Antibodies used in flow cytometry are listed in Table S2.

### 2.14 Giemsa Staining

Isolated neutrophils (1 × 10^6^) were collected on cytoslides using cytospin centrifugation. The cells were fixed with methanol and stained using diluted Giemsa solution (Sigma‐Aldrich). After the stained cells were rinsed in deionized water, the samples were observed under a light microscope (Leica DM750).

### 2.15 CYTO‐ID and Confocal Microscopy

Isolated neutrophils were subjected to staining using the CYTO‐ID Autophagy Detection Kit (Enzo Life Sciences). The cells were incubated with CYTO‐ID reagent (diluted 1:500) in RPMI 1640 supplemented with 3% FBS for 30 min at 37°C. Subsequently, the cells were seeded at a concentration of 1 × 10^6^ cells per well in 24‐well plates (Thermo Fisher Scientific) with coverslips coated with poly‐L‐lysine (Sigma‐Aldrich). The cells were then fixed using 4% paraformaldehyde (Sigma‐Aldrich) in PBS for 30 min at room temperature. Nuclei were stained with Hoechst (diluted 1:2000; Thermo Fisher Scientific) for 5 min. The cover glasses were mounted onto glass slides and visualized using a Zeiss LSM700 microscope (Zeiss). The acquired images were subsequently processed with Zen software (Zeiss).

### 2.16 Quantitative RT‐PCR (qRT‐PCR)

Total RNA was isolated from neutrophils using TRIzol reagent (Life Technology, Carlsbad, CA, USA) according to the manufacturer's protocol. Isolated RNA was used to synthesize cDNA using a Maxime RT PreMix Kit (iNtRON Biotechnology, Seongnam, Korea) Quantitative polymerase chain reaction (qPCR) was performed with a Rotor‐Gene SYBR Green PCR Kit (BIOFACT, Daejeon, Korea) and gene‐specific primers: *Map1lc3b*‐forward, 5′‐ATCTGAGCAGAGGGAAAAGGG‐3′; *Map1lc3b*‐reverse, 5′‐GCTGTCCCGAATGTCTCCT‐3′; *Atg5*‐forward, 5′‐GTCCATCCAAGGATGCGGT‐3′; *Atg5*‐reverse, 5′‐ATTCTGCAGTCCCATCCAGAG‐3′; *Atg12*‐forward, 5′‐AACAAAGAAATGGGCTGTGG‐3′; *Atg12*‐reverse, 5′‐TTGCAGTAATGCAGGACCAG‐3′; *Gapdh*‐forward, 5′‐TCCACCACCCTGTTGCTGTA‐3′; and *Gapdh*‐reverse, 5′‐AATGTGTCCGTCGTGGATCT‐3′. For qPCR, 55 PCR cycles were performed in three steps including denaturation (95°C, 30 s), annealing (60°C, 30 s) and extension (72°C, 1 min). Relative gene expression levels were normalized to the *Gapdh* expression level.

### 2.17 Statistical Analysis

The results were evaluated via GraphPad Prism software. Data are presented as mean ± standard deviation (SD). The statistical analysis was performed using Student's *t*‐test or analysis of variance (ANOVA). Survival data were analysed using the log‐rank test. *p* ≤ 0.05 was considered statistically significant.

## 3 Results

### 3.1 FMM Promotes Antimicrobial Activity in Neutrophils While Simultaneously Suppressing Inflammatory Cytokine Production

**FIGURE 1:**
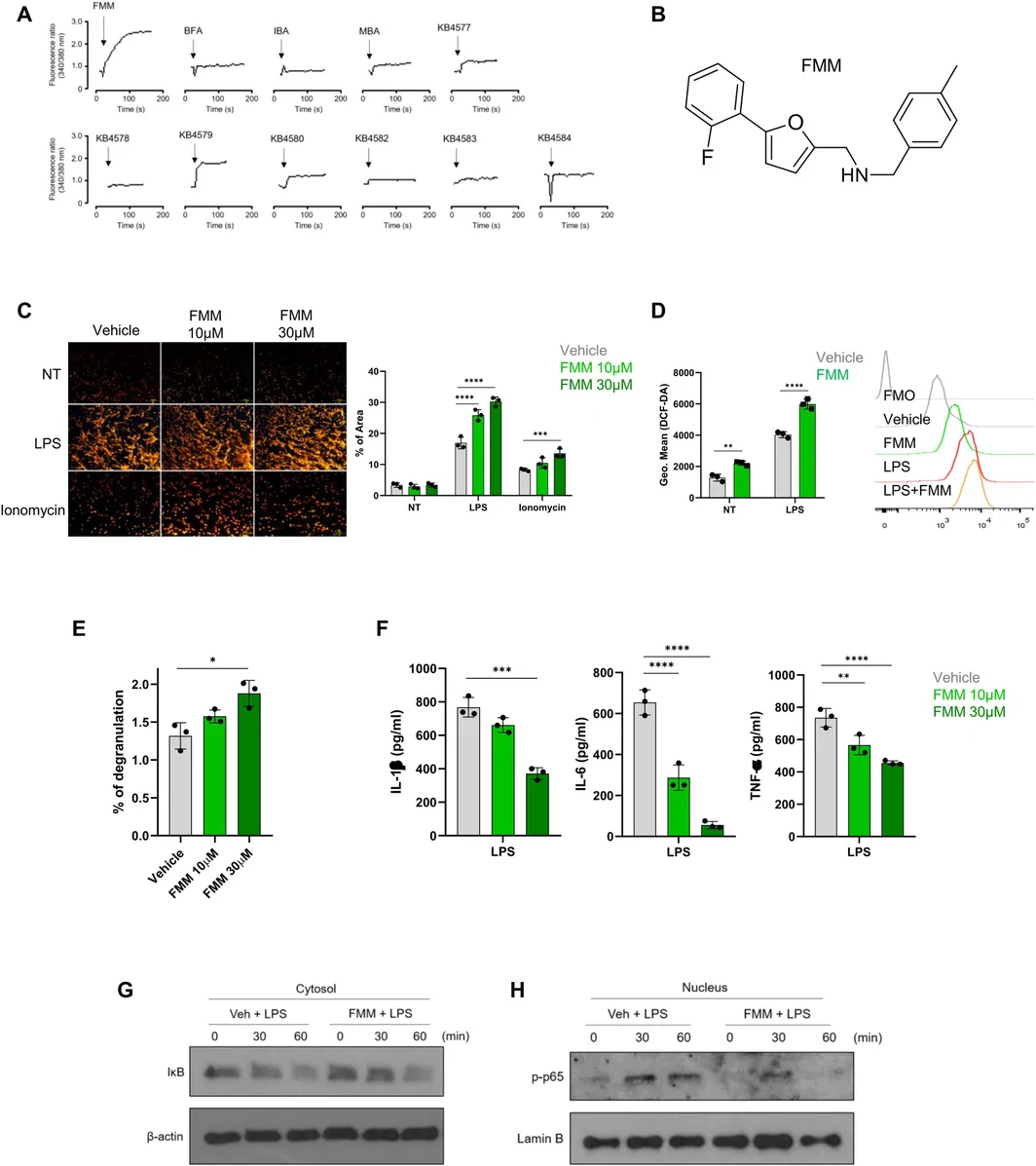
FMM enhances neutrophil antimicrobial functions while suppressing LPS‐induced pro‐inflammatory cytokine production. (A) Neutrophils were stimulated with several synthetic intermediates of sulfonamide, and intracellular Ca^2+^ levels were measured using a Fura‐2‐based assay. The fluorescence ratios (340/380 nm) were monitored after compound addition. (B) Compound structure of FMM. (C) Bone marrow neutrophils were stimulated with LPS (50 μg/mL) or ionomycin (5 μM) for 4 h in the presence of vehicle or FMM (10 and 30 μM). NET formation was measured using SYTOX Orange nucleic acid staining and visualized by fluorescence microscopy (left). Scale bar, 100 μm. The area of SYTOX Orange staining was quantified per microscopic field (right). (D) Bone marrow neutrophils were stimulated with or without 1 μg/mL of LPS in the presence of vehicle or FMM (30 μM). Cellular ROS was measured by DCF‐DA for 30 min. The bar graph indicates the quantified Geometric mean. Representative flow cytometry histograms of DCF‐DA expression on neutrophils. (E) FMM was administrated to neutrophils for the degranulation assay. (F) Neutrophils were stimulated with 1 μg/mL of LPS for 24 h in the presence of vehicle or FMM (10 and 30 μM). (G, H) Bone marrow neutrophils were stimulated with LPS (1 μg/mL) for 0, 30, or 60 min in the presence of vehicle or FMM (30 μM). After fractionation, cytosolic and nuclear fractions were separated on SDS‐PAGE, and the levels of p‐p65, IκB, β‐actin and Lamin B were determined by Western blot analysis. The data are expressed as the mean ± SD (*n* = 3–5). *p < 0.05; **p < 0.01; ***p < 0.001; ****p < 0.0001. Statistical significance was determined by two‐way ANOVA (C, D) and Student's *t*‐test (E, F).

Sulfonamides are typically synthesized via a nucleophilic substitution reaction between sulfonyl chlorides (R^1^SO_2_Cl) and amine‐containing compounds. We synthesized several intermediates of sulfonamide generation according to a previous report [27]. Newly synthesized intermediates include KB‐4577, KB‐4578, KB‐4579, KB‐4580, KB‐4582, KB‐4583, and KB‐4584 as shown in Figure S2. Given that neutrophils are crucial for innate immunity and their activation involves Ca^2+^ signalling [28], we investigated the effects of our newly synthesized sulfonamide intermediates on Ca^2+^ increase in these cells. Among the synthesized sulfonamide intermediates, FMM increased intracellular Ca^2+^ levels (Figure 1A,B), indicating its potential to enhance neutrophil functions. Although KB‐4579 increased the 340/380 ratio, spectrofluorophotometer pattern analysis demonstrated that this elevation was not attributable to a rise in calcium ion levels. Therefore, we examined whether FMM modulates neutrophil functions. NETs, composed of DNA and antimicrobial proteins, are one such defence mechanism that traps and eliminates pathogens [29]. Although FMM alone did not induce NET formation, it markedly enhanced NETs generation in the presence of LPS or ionomycin (Figure 1C). FMM enhanced neutrophil elastase release in a concentration‐dependent manner under both ionomycin and LPS stimulation (Figure S2). The production of ROS is a critical trigger for NET formation [30]. Neutrophils treated with FMM exhibited a concentration‐dependent increase in ROS production. Furthermore, under LPS‐stimulated conditions, FMM significantly augmented ROS levels compared to LPS alone (Figure 1D). Similarly, neutrophil degranulation was significantly elevated in FMM‐treated neutrophils (Figure 1E). However, FMM treatment did not affect neutrophil phagocytic activity (Figure S2). Collectively, these augmented antimicrobial mechanisms indicate that FMM strengthens neutrophil‐mediated host defence responses.

Excessive production of inflammatory cytokines is a primary driver of immune‐mediated tissue damage, making its reduction a crucial therapeutic objective [31]. To elucidate whether FMM modulates cytokine production in neutrophils, the impact of FMM on LPS‐induced inflammatory cytokine synthesis was investigated. FMM treatment significantly and concentration‐dependently attenuated LPS‐induced production of IL‐1β, IL‐6, and TNF‐α (Figure 1F). These inflammatory cytokines are produced following NF‐κB activation [32]. Under homeostatic conditions, NF‐κB remains inactive in the cytoplasm, sequestered by its inhibitor, IκB. Upon stimulation, IκB undergoes phosphorylation and subsequent proteasomal degradation, leading to NF‐κB activation [32]. To further delineate the mechanism underlying the anti‐inflammatory effects of FMM, the levels of IκB and p65 were analysed. LPS stimulation markedly reduced cytoplasmic IκB levels, whereas FMM treatment effectively attenuated this degradation (Figure 1G). Consistent with this, FMM also attenuated the LPS‐induced nuclear translocation of phosphorylated p65 (Figure 1H). These findings collectively indicate that FMM suppresses NF‐κB signalling by inhibiting IκB degradation and preventing p65 nuclear translocation, thereby contributing to the observed reduction in inflammatory cytokine production.

### 3.2 FMM Elicits Therapeutic Effect Against Sepsis

**FIGURE 2:**
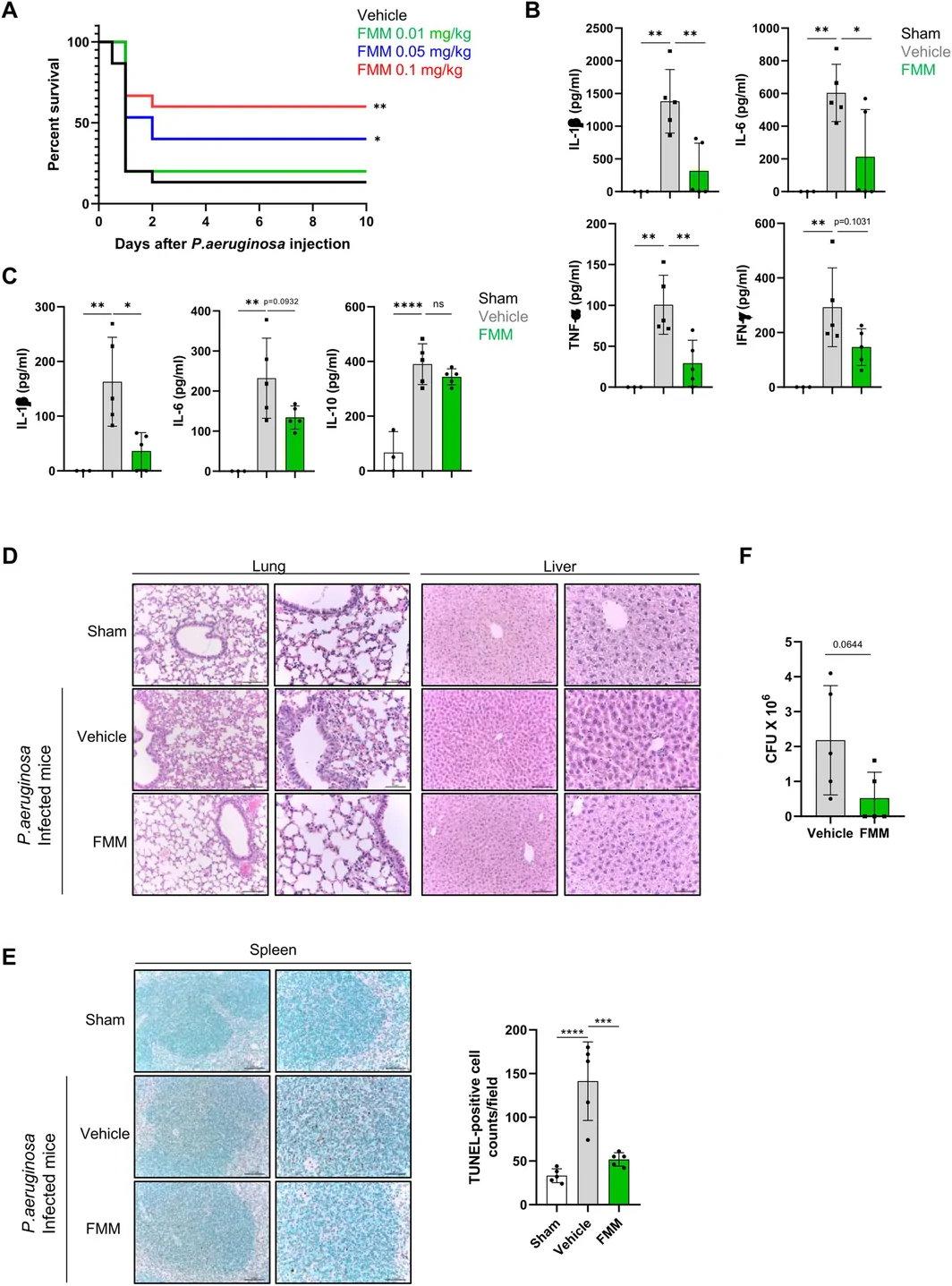
FMM elicits therapeutic effects in an experimental sepsis model. (A) FMM (0, 0.01, 0.05, 0.1 mg/kg) was administered four times at 12 h intervals from 2 h after infection. Survival was monitored for 10 days. *p < 0.05; **p < 0.01 compared to vehicle control by analysis of variance (ANOVA). *n* = 10 mice per group. (B–F) FMM (0.1 mg/kg) was administered into mice at 2 and 14 h after infection. The mice were sacrificed 24 h after infection. Cytokine levels in the peritoneal fluid (B) and serum (C) were determined by enzyme‐linked immunosorbent assay (ELISA). (D) Lungs and livers were stained with haematoxylin and eosin (Scale bar, 100 μm (left) 50 μm (right)). (E) A representative image of the TUNEL assay was performed on splenic tissue (Scale bar, 100 μm (left) 50 μm (right)). The quantified number of TUNEL^+^ cells was shown in the bar graph. (F) Colony‐forming units (CFUs) were determined from peritoneal lavage fluid. The data are expressed as the mean ± SD (*n* = 3–5). *p < 0.05; **p < 0.01; *** < 0.001; **** < 0.0001. Statistical significance was determined by one‐way ANOVA (B, C, E) or Student's *t*‐test (F).

Our in vitro findings revealed that FMM fine‐tunes neutrophil function, boosting key innate defence mechanisms while simultaneously limiting excessive inflammation. These promising results led us to investigate the therapeutic potential of FMM in an animal model of sepsis. In a *P. aeruginosa* ‐induced sepsis model, FMM administration significantly improved survival rates in a dose‐dependent manner. Specifically, administration of 0.1 mg/kg FMM increased survival to 60% in an experimental sepsis model, a substantial improvement over the 20% observed in the vehicle group (Figure 2A). Given that *P. aeruginosa* infection is known to induce an uncontrolled inflammatory response [33], we assessed the impact of FMM on inflammatory cytokine levels. FMM administration significantly attenuated the levels of TNF‐α, IL‐1β, and IL‐6 in the peritoneal lavage fluid, and IFN‐γ was slightly decreased (Figure 2B). In the serum, FMM decreased IL‐1β and IL‐6 levels, whereas IL‐10 levels remained unchanged compared with the vehicle (Figure 2C). Sepsis‐associated mortality in mice is closely linked to inflammation and dysfunction of vital organs, including the lungs and liver [34]. FMM treatment substantially alleviated lung injury induced by *P. aeruginosa* infection, as indicated by reduced alveolar wall congestion and cellular infiltration on histological examination (Figure 2D). Similarly, liver injury, such as sinusoidal obstruction and inflammation, was observed but markedly reduced by FMM treatment (Figure 2D). Furthermore, sepsis‐induced immune cell apoptosis contributes to organ dysfunction and poor outcomes [35]. TUNEL assay demonstrated increased splenocyte apoptosis in *P. aeruginosa* ‐infected mice, which was significantly diminished by FMM administration (Figure 2E). These findings indicate that FMM effectively elicits a therapeutic effect against sepsis by suppressing excessive inflammatory responses and immune cell apoptosis during infection.

To ascertain whether FMM contributes to bacterial clearance, bacterial loads at infection sites were quantified. In the early stages of sepsis, systemic inflammation and mortality are closely associated with defective bacterial clearance, particularly peritoneal bacterial colony counts [36]. While *P. aeruginosa* infection led to an increase in bacterial colony counts, FMM administration significantly reduced these counts compared to the vehicle group (Figure 2F), indicating enhanced in vivo bacterial clearance. In a separate experiment, it was observed that FMM did not directly inhibit bacterial growth in vitro (Fig. S3). These collective results suggest that the antibacterial effect of FMM is likely mediated through host immune modulation rather than direct bactericidal activity.

### 3.3 FMM Regulates Neutrophil Production in the Bone Marrow

**FIGURE 3:**
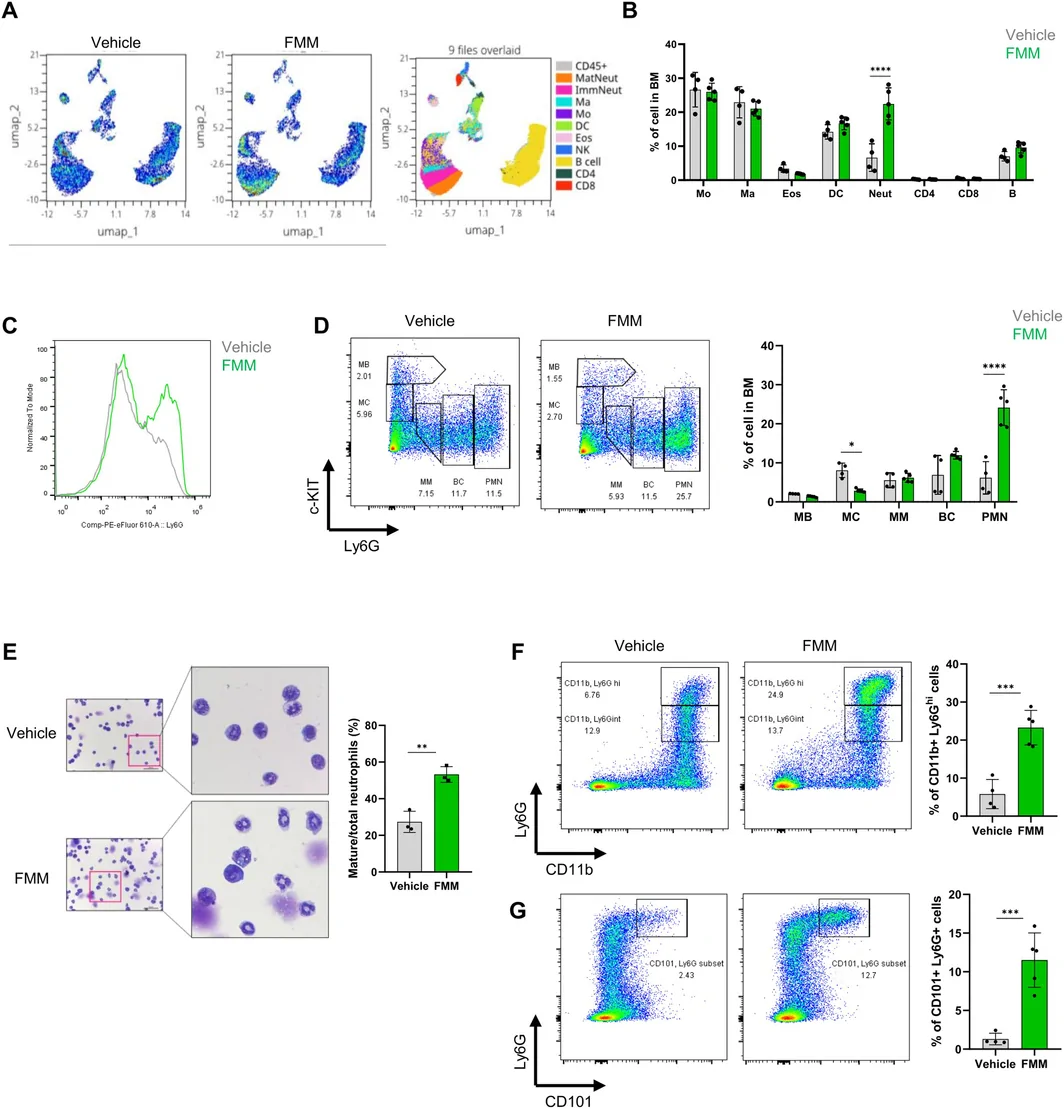
FMM enhances neutrophil maturation during sepsis. (A–G) FMM (0.1 mg/kg) was administered into mice at 2 and 14 h after infection. The mice were sacrificed 24 h after infection. (A) UMAP representation of total bone marrow CD45^+^ immune cells analysed using phenotyping by accelerated refined community‐partitioning clustering. Frequencies of immune cell populations, including mature neutrophils (MatNeut), immature neutrophils (ImmNeut), macrophages (Ma), dendritic cells (DC), eosinophils (Eos), NK cells, B cells, CD4^+^ T cells, and CD8^+^ T cells, were identified. (B) Percentage of immune cell subtypes within CD45^+^ cells in *P. aeruginosa* ‐infected mice. (C) Flow cytometry histogram of Ly6G expression on neutrophils. (D) Neutrophil development was assessed by measuring the expression of Ly6G and c‐kit in *P. aeruginosa* ‐infected mice. [Ly6G^−^ c‐kit^high^ myeloblasts (MB), Ly6G^−^ c‐kit^mid^ myelocytes (MC), Ly6G^low^ c‐kit^low^ metamyelocytes (MM), Ly6G^mid^ c‐kit^low^ band cells (BC), Ly6G^high^ c‐kit^low^ polymorphonuclear neutrophils (PMN)]. (E) Isolated bone marrow neutrophils from *P. aeruginosa* ‐infected mice were stained with Giemsa staining solution, and mature neutrophils (segmented neutrophils) were counted under a light microscope. (F) Representative flow cytometry analysis of Ly6G and CD11b expression in neutrophils from *P. aeruginosa* ‐infected mice. The bar graph indicates the percentage of Ly6G^hi^ neutrophil subsets. (G) Representative flow cytometry analysis of Ly6G and CD101 expression in neutrophils from *P. aeruginosa* ‐infected mice. The bar graph indicates the percentage of CD101^+^ neutrophil subsets. The data are expressed as the mean ± SD (*n* = 3–5). *p < 0.05; **p < 0.01; ***p < 0.001; ****p < 0.0001. Statistical significance was determined by two‐way ANOVA (B, D) and Student's *t*‐test (E–G).

Sepsis disrupts bone marrow haematopoiesis by inducing emergency granulopoiesis, which leads to the release of immature and functionally impaired neutrophils [37]. This dysregulated neutrophil production not only impairs bacterial clearance but also contributes to the overall immunocompromised state observed in sepsis patients [38]. Thus, the immune cell profiles in the bone marrow of mice treated with either vehicle or FMM were analysed after *P. aeruginosa* infection. UMAP dimensionality reduction was used to visualize and compare the distribution of major immune cell populations, including monocytes (Mo), macrophages (Ma), eosinophils (Eos), dendritic cells (DC), neutrophils (Neut), T cells (CD4, CD8), and B cells (Figure S4). UMAP analysis of bone marrow immune cells revealed distinct clustering. Notably, FMM administration led to a selective expansion of the mature neutrophil (MatNeut) population compared to the vehicle (Figure 3A). Quantitative analysis confirmed that FMM administration significantly increased neutrophil frequencies (Figure 3B), whereas other myeloid and lymphoid populations showed no significant differences (Figure 3B). Furthermore, Ly6G expression was elevated in bone marrow cells from FMM‐administrated mice (Figure 3C). The effects of FMM on bone marrow neutrophil development during sepsis were examined. Neutrophil differentiation was assessed by analysing c‐Kit and Ly6G expression levels using a previously described gating strategy [39]. FMM administration significantly increased the population of polymorphonuclear neutrophils (PMN) in the bone marrow while decreasing myelocytes (MC). No significant changes were observed in other neutrophil developmental stages, including myeloblasts (MB), metamyelocytes (MM), and band cells (BC) (Figure 3D). Giemsa staining further confirmed a significant increase in segmented mature neutrophils in FMM‐administrated mice (Figure 3E). The frequency of Ly6G^+^ and CD101^+^ mature neutrophils was significantly increased in FMM‐treated mice compared to the vehicle (Figure 3F,G). Collectively, these data demonstrate that FMM promotes neutrophil maturation within the bone marrow during septic condition.

### 3.4 FMM Enhances Neutrophil Autophagy

**FIGURE 4:**
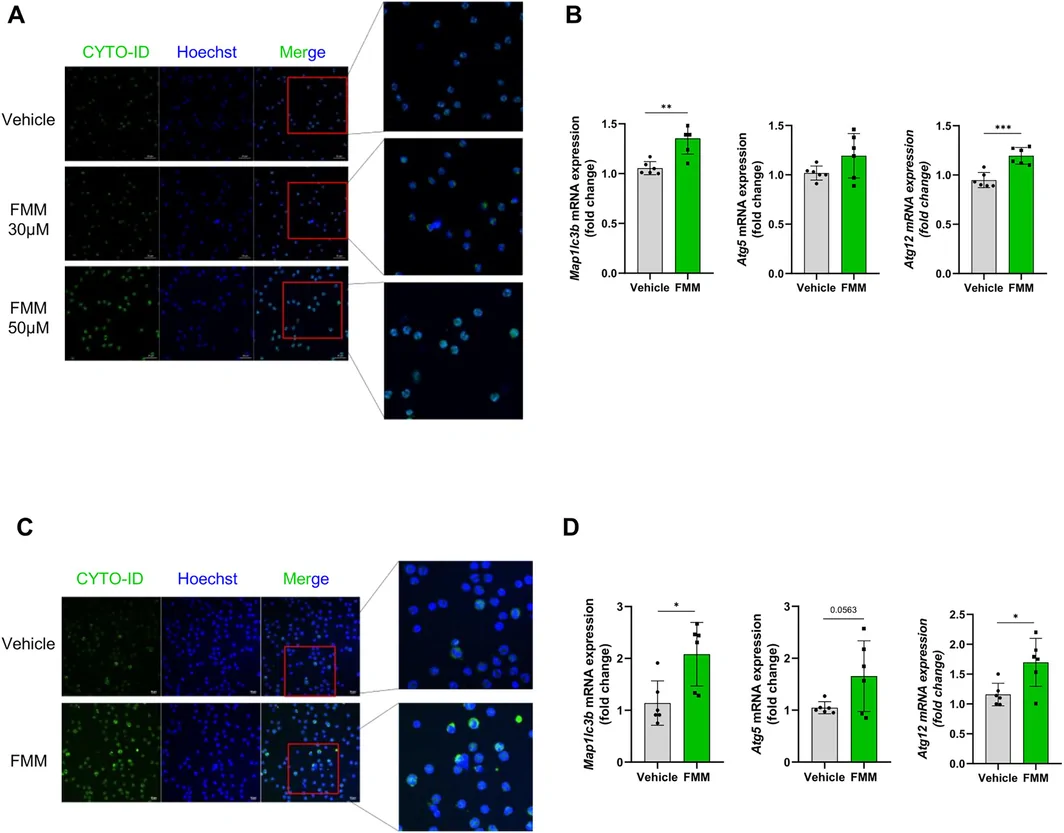
FMM promotes neutrophil autophagy. (A) Bone marrow neutrophils were treated with vehicle or FMM (30, 50 μM) for 6 h and then subjected to CYTO‐ID staining. The samples were subsequently analysed via confocal microscopy. Representative images displaying CYTO‐ID (green) and Hoechst (blue) are shown. Scale bar, 20 μm. (B) Neutrophils were stimulated with FMM for 6 h. Quantitative real‐time polymerase chain reaction (qPCR) was used to measure mRNA expression levels of autophagy‐related genes (*Map1lc3b, Atg5*, *Atg12*). (C) Isolated bone marrow neutrophils from *P. aeruginosa* ‐infected mice were stained with CYTO‐ID staining. The samples were subsequently analysed via confocal microscopy. Representative images displaying CYTO‐ID (green) and Hoechst (blue) are shown. Scale bar, 10 μm. (D) Isolated bone marrow neutrophils from *P. aeruginosa* ‐infected mice were analysed via qPCR. The data are expressed as the mean ± SD (*n* = 3–6). *p < 0.05; **p < 0.01; ***p < 0.001. Statistical significance was determined by Student's *t*‐test (B, D).

Autophagy, an intracellular degradation and energy‐recycling mechanism, is a crucial homeostatic process contributing to neutrophil differentiation and function [40, 41]. To elucidate the mechanism of FMM‐mediated neutrophil maturation, autophagic activity was analysed using CYTO‐ID staining, a fluorescent probe for specific visualization of autophagosomes in live cells [42]. FMM treatment increased CYTO‐ID positive cells in a concentration‐dependent manner in vitro (Figure 4A). Consistently, the mRNA levels of the autophagy‐related genes *Map1lc3b* and *Atg12* were significantly upregulated in FMM‐treated neutrophils (Figure 4B). To determine if FMM induces autophagy in vivo under inflammatory conditions, FMM was administered to *P. aeruginosa* ‐infected mice. Consistent with the in vitro findings, CYTO‐ID staining revealed a marked increase in autophagosome formation in neutrophils isolated from FMM‐administered mice (Figure 4C). Moreover, the expression of autophagy‐related genes was also upregulated in neutrophils from these mice (Figure 4D). Taken together, these results strongly suggest that FMM enhances autophagic activity in neutrophils.

### 3.5 FMM May Activate GPCR‐Mediated Signalling Pathways in Neutrophils

**FIGURE 5:**
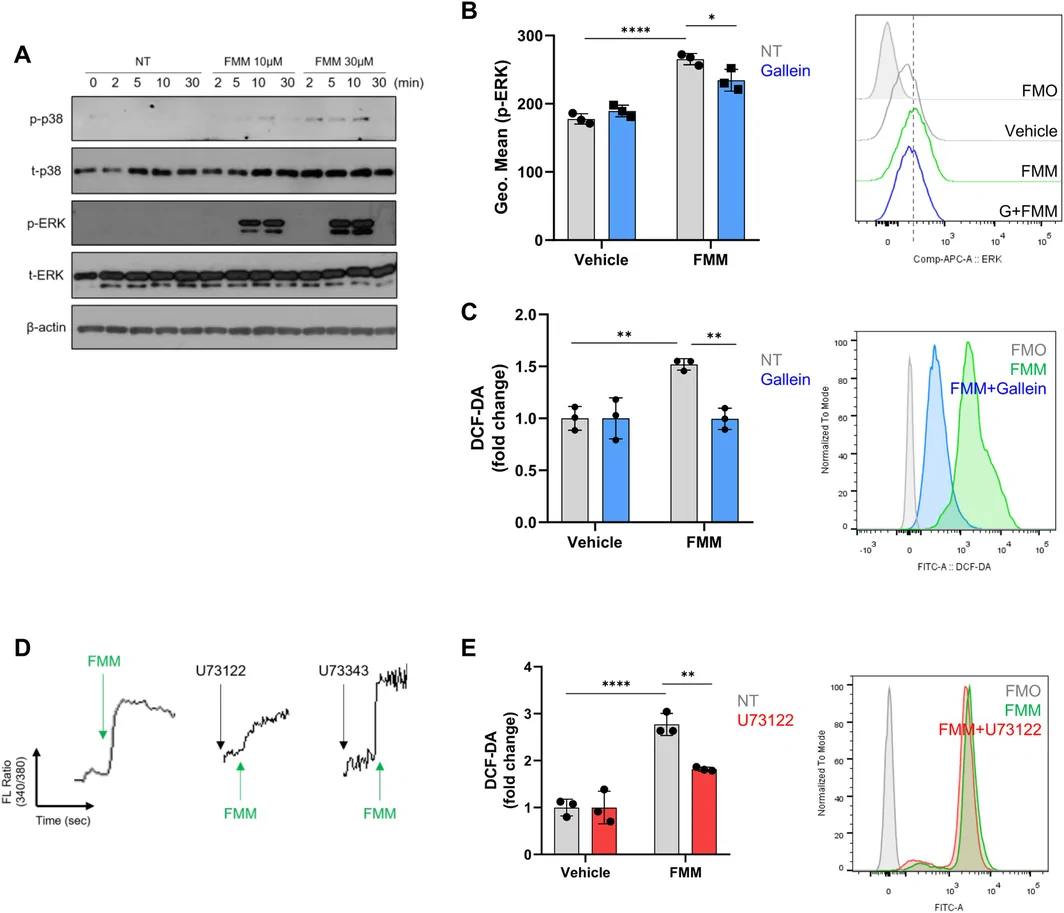
FMM stimulates G_βγ_‐PLC‐dependent signalling pathways in neutrophils. (A) Neutrophils were stimulated with FMM for various lengths of time. The levels of p‐p38, t‐p38, p‐ERK, t‐ERK, and β‐actin were measured using western blot analysis. (B, C) Cells were pretreated with a G_βγ_ inhibitor (Gallein 10 μM) for 30 min before FMM (30 μM) treatment. (B) Effect of G_βγ_ inhibition on FMM‐induced p‐ERK expression in neutrophils, as measured by flow cytometry. Representative flow cytometry histograms showing p‐ERK expression in neutrophils. (C) Effect of G_βγ_ inhibition on FMM‐induced ROS production measured by DCF‐DA fluorescence. Representative flow cytometry histograms of DCF‐DA expression on neutrophils. (D) Relative cytosolic Ca^2+^ concentrations were expressed as fluorescence ratios (340/380 nm). Fura‐2‐loaded neutrophils were stimulated with FMM (50 μM) in the absence or presence of U‐73122 (5 μM) or U‐73343 (5 μM). (E) Effect of a PLC inhibitor (U‐73122, 5 μM) on FMM‐induced ROS production measured by DCF‐DA fluorescence. Cells were pretreated with U‐73122 for 30 min before FMM (30 μM) treatment. The data are expressed as the mean ± SD (*n* = 3). *p < 0.05; **p < 0.01; ****p < 0.0001. Statistical significance was determined by two‐way ANOVA.

To elucidate the molecular mechanism by which FMM modulates neutrophil activation and exerts therapeutic effects in sepsis, the impact of FMM on key neutrophil signalling pathways, including MAPK (ERK and p38) activation and intracellular calcium increase. FMM rapidly induced the phosphorylation of ERK and p38 (Figure 5A), a transient activation profile consistent with G protein‐coupled receptor (GPCR)‐mediated signalling [43, 44]. To examine the involvement of G protein signalling, cells were pretreated with Gallein, a selective G_βγ_ inhibitor. Gallein pretreatment significantly suppressed FMM‐induced ERK phosphorylation (Figure 5B). Furthermore, the FMM‐induced increase in ROS production was also markedly attenuated by Gallein treatment (Figure 5C). The results suggest that FMM may activate neutrophils via GPCR.

To further investigate the downstream signalling of G_βγ_, the activation of phospholipase C (PLC), which plays a crucial role in regulating intracellular calcium levels [45]. Analysis of intracellular calcium revealed that FMM treatment induced a rapid calcium increase. Importantly, this calcium increase was significantly attenuated by pretreatment with U‐73122, a PLC inhibitor, whereas its inactive analog U‐73343 had no effect (Figure 5D). Consistently, U‐73122 pretreatment also partially reduced FMM‐induced ROS generation (Figure 5E). These results collectively suggest that FMM‐induced neutrophil activation is mediated through a G_βγ_‐PLC‐dependent signalling pathway.

## 4 Discussion

Despite advances in critical care, sepsis remains a major cause of mortality worldwide, largely due to its complex immunopathology encompassing both hyperinflammation and immune suppression [1, 2]. Neutrophil dysfunction is a central feature of this deregulation, leading to impaired pathogen clearance and tissue damage [46]. In this study, we identified FMM, a synthetic intermediate in sulfonamide synthesis, as a promising immunomodulator that enhances neutrophil maturation and antimicrobial function while attenuating inflammation, thereby improving survival in a *P. aeruginosa* ‐induced sepsis model.

The therapeutic efficacy of FMM suggests that sulfonamide intermediates may possess overlooked immunomodulatory activity. Sulfonamide‐based compounds have been reported to modulate immune responses, further highlighting their potential in immunotherapy. Interestingly, sulfonamides can selectively impair regulatory T cells, thereby potentially enhancing anti‐tumour immune responses [47]. In addition, sulfonamide compounds have also been reported to promote NET formation through lipid oxidation. Sulfasalazine enhanced NET formation by accelerating oxidation of ether‐linked phospholipids [24]. As a synthetic intermediate in sulfonamide synthesis, FMM does not exhibit direct antibacterial activity but appears to promote immunomodulatory responses, offering a novel approach to modulate host defence. Given that FMM is a sulfonamide synthesis intermediate containing biologically active moieties, it is possible that such intermediates themselves represent a previously unexplored class of immune‐modulating agents.

Neutrophil maturation is a crucial therapeutic target in sepsis [46], where emergency granulopoiesis produces immature, dysfunctional neutrophils that indicate disease severity [48]. These dysfunctional neutrophils exhibit functional immunosuppression despite apparent leukocytosis [49]. Unlike conventional anti‐inflammatory agents that broadly suppress immune activity [50], FMM promotes neutrophil maturation while attenuating inflammation. FMM enhanced neutrophil maturation and promoted autophagy. Based on established studies demonstrating that autophagy is crucial for neutrophil differentiation and antimicrobial function [40, 41], the findings suggest that FMM‐induced autophagy may contribute to the enhanced neutrophil maturation. Previously, it was demonstrated that administration of α‐CD200R antibodies improved outcomes in septic models by counteracting CD200‐mediated suppression of neutrophil autophagy and maturation [39]. Similarly, taurolidine and simvastatin enhance host defence and alleviate inflammation in sepsis through autophagy induction [51, 52]. The ability of FMM to drive beneficial neutrophil autophagy suggests its therapeutic potential against sepsis.

Although promoting neutrophil autophagy and ROS production may lead to host tissue damage, the overall effect of FMM in our model was clearly protective. Neutrophils are well‐recognized as double‐edged mediators of immunity, essential for pathogen clearance, yet capable of causing collateral tissue damage through excessive release of ROS, proteases, and NETs. However, whether neutrophil activation is beneficial or harmful critically depends on the context and degree of activation. In support of this notion, previous work demonstrated that enhanced NET formation and neutrophil antimicrobial function achieved by targeting phospholipase D2 improved survival and reduced organ damage in experimental sepsis without causing immunopathology [53]. Consistent with this, FMM administration in the present study reduced lung and liver tissue injury (Figure 2D) and decreased splenocyte apoptosis (Figure 2E), despite enhancing neutrophil ROS production, NET formation, and maturation. Furthermore, FMM suppressed excessive pro‐inflammatory cytokine production via NF‐κB inhibition (Figure 1F–H), indicating that FMM drives a balanced and appropriate immune activation rather than uncontrolled neutrophil hyperactivation.

Persistent exposure to inflammatory cytokines and pathogens during sepsis drives immune cell exhaustion and apoptosis, contributing to immunosuppression [35]. It was observed that the therapeutic effect of FMM in sepsis correlated with reduced levels of pro‐inflammatory cytokines (Figure 2B,C) and decreased tissue damage in vital organs (Figure 2D). FMM administration also reduced splenocyte apoptosis, which is significant given that excessive immune cell apoptosis contributes to sepsis‐induced immunosuppression (Figure 2E). Moreover, FMM inhibited LPS‐induced NF‐κB activation by stabilizing IκB and preventing nuclear translocation of p65, thereby suppressing TNF‐α, IL‐1β, and IL‐6 expression. By inhibiting NF‐κB activity, compounds such as FMM can effectively attenuate the systemic inflammatory response that drives organ damage and mortality in sepsis. Considering its selective inhibition of NF‐κB, FMM represents a promising therapeutic approach for re‐establishing immune balance and enhancing sepsis outcomes.

The results of this study suggest that the biological activity of FMM is largely attributed to its unique structural features. The molecule contains a furan ring, a fluorophenyl group, and a benzylamine moiety, each known for distinct pharmacological properties. The fluorophenyl group is reported to enhance bioavailability and receptor binding affinity [54], whereas the benzylamine moiety has demonstrated anti‐inflammatory effects [55]. The furan ring, frequently found in bioactive compounds, is associated with antimicrobial and immunomodulatory functions [56, 57]. Importantly, these structural motifs correspond to established sulfonamide chemotypes that serve as privileged scaffolds for GPCR modulation [58, 59]. The furan moiety is recognized for facilitating GPCR interactions [60], whereas the sulfonamide group forms critical hydrogen bonds with conserved residues in GPCR binding pockets [59]. In this study, we were unable to definitively identify a direct molecular target of FMM that mediates its neutrophil activation and subsequent anti‐septic activity. Instead, mechanistic investigations revealed that FMM activated GPCR signalling via G_βγ_ and PLC, as evidenced by reduced FMM‐induced responses after Gallein (a G_βγ_ inhibitor) or U‐73122 (a PLC inhibitor) pretreatment. Consistent with these structural insights, the results of this study suggest that FMM exerts its effects through a GPCR‐mediated signalling pathway involving G_βγ_ and PLC. While we demonstrated that FMM activates GPCR‐mediated G_βγ_‐PLC signalling and downstream pathways in vitro, it remains unclear whether this signalling is similarly regulated in neutrophils under septic conditions in vivo. Further studies are required to directly assess these pathways in neutrophils isolated from infected animals. Although the specific receptor remains unidentified, the presence of structural motifs with proven GPCR‐binding potential indicates this pathway as a mechanistically plausible target for future therapeutic development.

There are some limitations to this study. First, while we utilized LPS as a surrogate for certain in vitro experiments, it may not fully replicate the multifaceted immune response triggered by live *P. aeruginosa* . Second, although our systemic sepsis model utilized intraperitoneal injection to induce multi‐organ failure, future studies using intratracheal administration would be beneficial to specifically model *P. aeruginosa*‐induced pneumonia‐derived sepsis.

Collectively, these findings identify FMM as a previously uncharacterized sulfonamide‐derived immunomodulator that exerts therapeutic efficacy in sepsis through autophagy activation and GPCR‐mediated signalling. By promoting neutrophil maturation, enhancing antimicrobial activity, and limiting hyperinflammation, FMM restores immune balance and improves sepsis outcomes.

## Author Contributions

**Ji Ye Park:** conceptualization, investigation, formal analysis, writing – original draft, data curation, writing – review and editing. **Yu Sun Jeong:** conceptualization, investigation, writing – review and editing. **Myeongsu Shin:** resources, investigation, writing – review and editing. **Jiwoo Choi:** investigation. **Jiwon Shin:** investigation. **Leezhi Kwon:** investigation. **Kanghyun Lyu:** resources, investigation. **Hwangi Jo:** resources, investigation. **Youngjoo Byun:** conceptualization, writing – review and editing, funding acquisition, supervision, resources. **JaeHyung Koo:** conceptualization, supervision, writing – review and editing, funding acquisition. **Yoe‐Sik Bae:** conceptualization, funding acquisition, writing – review and editing, supervision.

## Funding

This work was supported by the National Research Foundation of Korea, RS‐2020‐NR046219, RS‐2024‐00401422, RS‐2025‐20562972.

## Conflicts of Interest

J.Y.P., Y.S.J., M.S., J.C., Y.B., J.K., and Y.‐S.B. have patent applications pending related to this work.

## Supporting information


**Figure S1:** (A) Synthesis of FMM. (B) Synthesis of sulfonamide intermediates (KB‐4577, KB‐4578, KB‐4579, KB‐4580, KB‐4582, KB‐4583, and KB‐4584).
**Figure S2:** Effect of FMM on elastase production and phagocytic activity in neutrophils. (A) Bone marrow neutrophils were stimulated with lipopolysaccharide (LPS) (50 μg/mL) or ionomycin (5 μM) for 4 h in the presence of vehicle or FMM (10 and 30 μM). Neutrophil Elastase levels were determined by enzyme‐linked immunosorbent assay (ELISA). (B) Neutrophils were co‐incubated with pHrodo BioParticles for 2 h. Phagocytic cells with engulfed bacteria were analysed by flow cytometry. The data are expressed as the mean ± SD (*n* = 3). ***p < 0.001. Statistical significance was determined by two‐way ANOVA.
**Figure S3:** FMM does not directly inhibit *P. aeruginosa* growth. *P. aeruginosa* was treated with FMM at concentrations ranging from 3.125 to 100 μM. After 24 h incubation at 37°C, bacterial growth was assessed by measuring optical density at 600 nm (O.D.600). Imipenem (10 μg/mL) and untreated cultures were used as positive and negative controls, respectively. The data are expressed as the mean ± SD (*n* = 3). ****p < 0.0001. Statistical significance was determined by Student's t‐test.
**Figure S4:** Gating strategy for flow cytometry. Gating strategy for immune cell population. The representative image of flow cytometry demonstrates the gating process. Total cells were first gated on FSC‐A versus SSC‐A. Singlets were then selected using FSC‐A versus FSC‐H, followed by gating of live cells by excluding FVD‐positive cells (FVD⁻). Among live cells, CD45⁺ leukocytes were gated for further analysis. Neutrophils were identified as Ly6G⁺ CD11b⁺ cells. The Ly6G⁻ CD11b⁺ population was further analysed to identify monocytes (CD11b+ Ly6C⁺), dendritic cells (CD11b− CD11c⁺) and other myeloid subsets. Within the Ly6G⁻ gate, F4/80⁺ populations were separated and further subdivided. Macrophages were identified as SiglecF⁻ CD11c⁺, and eosinophils as SiglecF⁺ CD11b⁺. In parallel, from the CD45⁺ population, CD3e⁺ T cells were gated and further separated into CD4⁺ and CD8⁺ T cell subsets. B cells were identified as CD19⁺ CD3e⁻ cells.

**Table S1:** List of antibodies used in Western blot.
**Table S2:** List of antibodies used in flow cytometry.

## Data Availability

The data that support the findings of this study are available from the corresponding author upon reasonable request.
