# Supplementary material for: A Sulfonamide Intermediate Elicits Therapeutic Effect Against Sepsis by Enhancing Neutrophil Maturation
Source: J Cell Mol Med. 2026 Sep 4;30(17):e71346. doi: 10.1111/jcmm.71346 (PMC13545274; doi:10.1111/jcmm.71346)
Supplement: Supplementary file 1 [file JCMM-30-e71346-s001.docx]

**A sulfonamide intermediate elicits therapeutic effect against sepsis by enhancing neutrophil maturation**

**Supplementary figures**

**Figure S1-A. Synthesis of FMM**

**Figure S1-B. Synthesis of sulfonamide intermediates (KB-4577, KB-4578, KB-4579, KB-4580, KB-4582, KB-4583, and KB-4584)**

General Procedure A: reductive amination of aldehyde with amines

To a solution of appropriate aldehyde (1.0 equiv) in methanol (5 mL) was added appropriate amine (1.0 equiv). The reaction mixture was stirred at room temperature for 1 ~ 2 h and cooled at 0°C. NaBH_4_ (3.0 equiv) was added to the reaction mixture with stirring. The mixture was stirred at room temperature until complete conversion monitored by TLC analysis, quenched by saturated aqueous NaHCO_3_ solution, and the excess solvent was removed under vacuum. The residue was diluted with EtOAc and the organic layer was washed with water and brine, dried over anhydrous MgSO_4_, and concentrated in vacuo. The crude was purified by column chromatography on a silica gel (Hexane/EtOAc) to afford the desired product.

*N-((5-Bromofuran-2-yl)methyl)-2-methylpropan-1-amine* (**KB-4577**)

Compound (**KB-4577**) was prepared in 89% yield (1.18 g) as a colorless liquid, following the same procedure as described in the general procedure A with 5-bromofuran-2-carbaldehyde (**1**) (1.0 g, 5.71 mmol) and isobutylamine (630 µL, 6.28 mmol). *R*_f_ = 0.30 (Hexane/EtOAc = 5:1); ^1^H NMR (600 MHz, CDCl_3_) δ 6.22 (d, *J* = 3.2 Hz, 1H), 6.15 (d, *J* = 3.2 Hz, 1H), 3.73 (s, 2H), 2.41 (d, *J* = 6.7 Hz, 2H), 1.78 – 1.70 (m, 1H), 0.91 (d, *J* = 6.7 Hz, 6H); HRMS (ESI) m/z calculated for C_9_H_15_BrNO^+^ [M + H]^+^: 232.0332; found: 232.0330; C_5_H_4_BrO^+^ [M – C_4_H_10_N]^+^: 158.9440; found: 158.9430.

*1-(5-Bromofuran-2-yl)-N-(4-methylbenzyl)methanamine* (**KB-4583**)

Compound (**KB-4583**) was prepared in 86% yield (137 mg) as a colorless liquid, following the same procedure as described in the general procedure A with 5-bromofuran-2-carbaldehyde (**1**) (100 mg, 0.57 mmol) and *p*-tolylmethanamine (73 µL, 0.57 mmol). *R*_f_ = 0.1 (Hexane/EtOAc = 9:1); ^1^H NMR (600 MHz, CDCl_3_) δ 7.21 (d, *J* = 7.9 Hz, 2H), 7.14 (d, *J* = 7.9 Hz, 2H), 6.23 (d, *J* = 3.2 Hz, 1H), 6.16 (d, *J* = 3.2 Hz, 1H), 3.75 (s, 2H), 3.74 (s, 2H), 2.34 (s, 3H); HRMS (ESI) m/z calculated for C_13_H_15_BrNO^+^ [M + H]^+^: 280.0332; found: 280.0355.

General Procedure B: the sulfonylation of amines

To a solution of appropriate amine in dichloromethane (5 mL) was added TEA or DIPEA (1.0 equiv) and 2-chlorobenzenesulfonyl chloride (0.9 ~ 1.2 equiv). The reaction mixture was stirred at room temperature for 12 h. The reaction mixture was quenched by water, and extracted with dichloromethane. The organic layer was washed with brine, dried with anhydrous MgSO_4_, and concentrated in vacuo. The crude was purified by column chromatography on a silica gel (Hexane/EtOAc) to afford the desired product.

*2-Chloro-N-((5-(2-fluorophenyl)furan-2-yl)methyl)-N-(4-methylbenzyl)benzenesulfonamide* (**KB-4582**)

Compound (**KB-4582**) was prepared in 73% yield (113 mg) as a colorless liquid, following the same procedure as described in the general procedure B with 1-(5-(2-fluorophenyl)furan-2-yl)-N-(4-methylbenzyl)methanamine (**FMM**) (110 mg, 0.37 mmol) and 2-chlorobenzenesulfonyl chloride (46 µL, 0.33 mmol). *R*_f_ = 0.40 (Hexane/EtOAc = 9:1); ^1^H NMR (600 MHz, CDCl_3_) δ 8.14 (d, *J* = 7.2 Hz, 1H), 7.49 – 7.42 (m, 3H), 7.38 – 7.34 (m, 1H), 7.25 – 7.20 (m, 1H), 7.18 (d, *J* = 8.0 Hz, 2H), 7.16 – 7.13 (m, 3H), 7.09 (ddd, *J* = 11.1, 7.2, 2.0 Hz 1H), 6.65 (t, *J* = 3.3 Hz, 1H), 6.12 (d, *J* = 3.3 Hz, 1H), 4.56 (s, 2H), 4.39 (s, 2H), 2.33 (s, 3H); HRMS (ESI) m/z calculated for C_25_H_21_ClFNO_3_SNa^+^ [M + Na]^+^: 492.0807; found: 492.0828.

*N-((5-Bromofuran-2-yl)methyl)-2-chloro-N-isobutylbenzenesulfonamide* (**KB-4578**)

Compound (**KB-4578**) was prepared in 91% yield (477 mg) as a colorless liquid, following the same procedure as described in the general procedure B with *N*-((5-bromofuran-2-yl)methyl)-2-methylpropan-1-amine (3) (300 mg, 1.29 mmol) and 2-chlorobenzenesulfonyl chloride (210 µL, 1.55 mmol). *R*_f_ = 0.33 (Hexane/EtOAc = 10:1); ^1^H NMR (600 MHz, CDCl_3_) δ 8.09 (d, *J* = 8.1 Hz, 1H), 7.51 – 7.45 (m, 2H), 7.39 – 7.35 (m, 1H), 6.13 (d, *J* = 3.2 Hz, 1H), 6.07 (d, *J* = 3.2 Hz, 1H), 4.41 (s, 2H), 3.15 (d, *J* = 6.6 Hz, 2H), 1.95 – 1.86 (m, 1H), 0.86 (d, *J* = 6.6 Hz, 6H); HRMS (ESI) m/z calculated for C_15_H_17_BrClNO_3_SNa^+^[M + Na]^+^: 427.9693; found: 427.9685; C_5_H_4_BrO^+^ [M – C_10_H_13_ClNO_2_S]^+^: 158.9440; found: 158.9426.

*N-((5-Bromofuran-2-yl)methyl)-2-chloro-N-(4-methylbenzyl)benzenesulfonamide* (**KB-4584**)

Compound (**KB-4584**) was prepared in 51% yield (76 mg) as a white solid, following the same procedure as described in the general procedure B with 1-(5-Bromofuran-2-yl)-N-(4-methylbenzyl)methanamine (**KB-4583**) (103 mg, 0.37 mmol) and 2-chlorobenzenesulfonyl chloride (46 µL, 0.33 mmol). *R*_f_ = 0.40 (Hexane/EtOAc = 9:1); ^1^H NMR (600 MHz, CDCl_3_) *δ* 8.12 (dd, *J* = 7.9, 1.3 Hz, 1H), 7.51 (dtd, *J* = 9.2, 7.9, 1.3 Hz, 2H), 7.38 (td, *J* = 7.9, 1.3 Hz, 1H), 7.16 – 7.12 (m, 4H), 6.10 (d, *J* = 3.2 Hz, 1H), 5.99 (d, *J* = 3.2 Hz, 1H), 4.53 (s, 2H), 4.25 (s, 2H), 2.34 (s, 3H); HRMS (ESI) m/z calculated for C_19_H_17_BrClNO_3_SNa^+^ [M + Na]^+^: 475.9693; found: 475.9719; C_5_H_4_BrO^+^ [M – C_14_H_13_ClNO_2_S]^+^: 158.9440; found: 158.9452.

General Procedure C: Suzuki coupling reactions

A mixture of the appropriate bromofuran substrate (1.0 equiv), Na_2_CO_3_ (2.0 equiv), and tetrakis(triphenylphosphine)palladium (0.02 equiv) in 1,2-dimethoxyethane (10 mL) and water (5 mL) was purged with argon. The reaction mixture was sonicated at room temperature for 5 min, and an appropriate boronic acid (1.25 equiv) in ethanol (2 mL) was added. The reaction mixture was stirred at 90 °C for 90 min. The reaction mixture was cooled to room temperature, and the residue was diluted with EtOAc. The organic layer was washed with aqueous NaHCO_3_ solution and brine, dried over anhydrous MgSO_4_, and concentrated in vacuo. The crude was purified by column chromatography on a silica gel (Hexane/EtOAc) to afford the desired product.

*2-Methyl-N-((5-(3-(methylsulfonyl)phenyl)furan-2-yl)methyl)propan-1-amine* (**KB-4580**)

Compound (**KB-4580**) was prepared in 49% yield (129 mg) as a pale orange liquid, following the same procedure as described in the general procedure C with *N*-((5-bromofuran-2-yl)methyl)-2-methylpropan-1-amine (**KB-4577**) (200 mg, 0.86 mmol) and (3-(methylsulfonyl)phenyl)boronic acid (220 mg, 1.08 mmol). *R*_f_ = 0.2 (DCM/MeOH = 97:3); ^1^H NMR (600 MHz, CDCl_3_) δ 8.20 (t, *J* = 1.6 Hz, 1H), 7.91 – 7.88 (m, 1H), 7.78 (ddd, *J* = 7.8, 1.6, 1.1 Hz, 1H), 7.56 (t, *J* = 7.8 Hz, 1H), 6.72 (d, *J* = 3.3 Hz, 1H), 6.31 (d, *J* = 3.3 Hz, 1H), 3.85 (s, 2H), 3.09 (s, 3H), 2.48 (d, *J* = 6.8 Hz, 2H), 1.81 – 1.74 (m, 1H), 0.93 (d, *J* = 6.8 Hz, 6H); HRMS (ESI) m/z calculated for C_16_H_22_NO_3_S^+^ [M + H]^+^: 308.1315; found: 308.1312; C_12_H_11_O_3_S^+^ [M – C_4_H_10_N]^+^: 235.0423; found: 235.0423.

*2-Chloro-N-isobutyl-N-((5-(3-(methylsulfonyl)phenyl)furan-2-yl)methyl)benzenesulfonamide* (**KB-4579**)

Compound (**KB-4579**) was prepared in 90% yield (212 mg) as a white solid, following the same procedure as described in the general procedure C with *N*-((5-bromofuran-2-yl)methyl)-2-chloro-*N*-isobutylbenzenesulfonamide (**KB-4578**) (200 mg, 0.49 mmol) and (3-(methylsulfonyl)phenyl)boronic acid (120 mg, 0.61 mmol). *R*_f_ = 0.35 (Hexane/EtOAc = 3:1); ^1^H NMR (600 MHz, CDCl_3_) δ 8.13 (dd, *J* = 7.9, 1.6 Hz, 1H), 8.07 (t, *J* = 1.7 Hz, 1H), 7.82 – 7.79 (m, 1H), 7.78 – 7.75 (m, 1H), 7.56 (t, *J* = 7.8 Hz, 1H), 7.49 (dd, *J* = 7.8, 1.3 Hz, 1H), 7.45 (ddd, *J* = 7.9, 7.6, 1.3 Hz,1H), 7.38 (ddd, *J* = 7.8, 7.6, 1.6 Hz, 1H), 6.64 (d, *J* = 3.3 Hz, 1H), 6.26 (d, *J* = 3.3 Hz, 1H), 4.54 (s, 2H), 3.17 (d, *J* = 6.7 Hz, 2H), 3.09 (s, 3H), 1.99 – 1.92 (m, 1H), 0.86 (d, *J* = 6.7 Hz, 6H); HRMS (ESI) m/z calculated for C_22_H_24_ClNO_5_S_2_Na^+^ [M + Na]^+^: 504.0676; found: 504.0675.

**2**

**FMM**

**KB-4577**

**KB-4583**

**KB-4578**

**KB-4582**

**KB-4580**

**KB-4584**

**KB-4579**

**Figure S2. Effect of FMM on elastase production and phagocytic activity in neutrophils.**

(A) Bone marrow neutrophils were stimulated with lipopolysaccharide (LPS) (50 μg/ml) or ionomycin (5 μM) for 4 h in the presence of vehicle or FMM (10 and 30 μM). Neutrophil Elastase levels were determined by enzyme-linked immunosorbent assay (ELISA). (B) Neutrophils were co-incubated with pHrodo BioParticles for 2 h. Phagocytic cells with engulfed bacteria were analyzed by flow cytometry. The data are expressed as the mean ± SD (n = 3). **p* < 0.05; ***p* < 0.01; ****p* < 0.001. Statistical significance was determined by two-way ANOVA.

(μM)

FMM

**Figure S3. FMM does not directly inhibit P. aeruginosa growth.**

*P. aeruginosa* was treated with FMM at concentrations ranging from 3.125 to 100 μM. After 24 h incubation at 37°C, bacterial growth was assessed by measuring optical density at 600 nm (O.D.600). Imipenem (10 μg/mL) and untreated cultures were used as positive and negative controls, respectively. The data are expressed as the mean ± SD (n = 3). *****p* < 0.0001. Statistical significance was determined by Student’s *t*-test.

**Figure S4. Gating strategy for flow cytometry.**

Gating strategy for immune cell population. The representative image of flow cytometry demonstrates the gating process. Total cells were first gated on FSC-A versus SSC-A. Singlets were then selected using FSC-A versus FSC-H, followed by gating of live cells by excluding FVD-positive cells (FVD⁻). Among live cells, CD45⁺ leukocytes were gated for further analysis. Neutrophils were identified as Ly6G⁺ CD11b⁺ cells. The Ly6G⁻ CD11b⁺ population was further analyzed to identify monocytes (CD11b^+^ Ly6C⁺), dendritic cells (CD11b^-^ CD11c⁺) and other myeloid subsets. Within the Ly6G⁻ gate, F4/80⁺ populations were separated and further subdivided. Macrophages were identified as SiglecF⁻ CD11c⁺, and eosinophils as SiglecF⁺ CD11b⁺. In parallel, from the CD45⁺ population, CD3e⁺ T cells were gated and further separated into CD4⁺ and CD8⁺ T cell subsets. B cells were identified as CD19⁺ CD3e⁻ cells.
