# Supplementary material for: A Sulfonamide Intermediate Elicits Therapeutic Effect Against Sepsis by Enhancing Neutrophil Maturation
Source: J Cell Mol Med. 2026 Sep 4;30(17):e71346. doi: 10.1111/jcmm.71346 (PMC13545274; doi:10.1111/jcmm.71346)
Supplement: Supplementary file 2 [file JCMM-30-e71346-s002.docx]

**A sulfonamide intermediate elicits therapeutic effect against sepsis by enhancing neutrophil maturation via autophagy**

**Supplementary Tables**

**Supplementary Table 1. List of antibodies used in Western blot.**

| Name | Source | Catalog no. | Dilution |
| --- | --- | --- | --- |
| p-p65 | SANTA CRUZ | sc-166748 | 1:1000 |
| IκBα | Cell signaling Technology | 4812 | 1:1000 |
| p-p38 | Cell signaling Technology | 9211S | 1:1000 |
| t-p38 | Cell signaling Technology | 9212S | 1:1000 |
| p-ERK | Cell signaling Technology | 9101S | 1:1000 |
| t-ERK | Cell signaling Technology | 9102S | 1:1000 |
| β-actin | Cell signaling Technology | 3700S | 1:1000 |
| LaminB1 | Abcam | Ab16048 | 1:1000 |
| HRP-linked  anti-rabbit IgG | Enzo Life Sciences | ADI-SAB-300-J | 1:5000 |
| HRP-linked  anti-mouse IgG | SeraCare | 5220-0342 | 1:5000 |

**Supplementary Table 2. List of antibodies used in flow cytometry.**

| Name | Source | Catalog no. | Dilution |
| --- | --- | --- | --- |
| Fixable Viability Dye eFluor^TM^ 780 | Thermo Fisher Scientific | 65-0865-14 | 1:1000 |
| Zombie NIR^TM^ | BioLegend | 423106 | 1:1000 |
| Ly6G | Thermo Fisher Scientific | 61-9668-82 | 1:200 |
| Siglec-F | Thermo Fisher Scientific | 14-1702-82 | 1:100 |
| CD45 | Thermo Fisher Scientific | 58-0451-82 | 1:100 |
| MHCII | Cytekbio | 60-5321 | 1:1000 |
| CD11b | Cytekbio | 75-0112 | 1:500 |
| NK1.1 | Cytekbio | 20-5941 | 1:100 |
| CD4 | Thermo Fisher Scientific | 67-0041-82 | 1:100 |
| CD3e | Thermo Fisher Scientific | 56-0032-82 | 1:100 |
| CD19 | Thermo Fisher Scientific | 414-0193-82 | 1:100 |
| CD101 | Thermo Fisher Scientific | 12-1011-82 | 1:100 |
| Ly6C | BioLegend | 128041 | 1:200 |
| F4/80 | BioLegend | 15-4801 | 1:500 |
| c-kit | BioLegend | 105828 | 1:100 |
| CD11c | BioLegend | 565586 | 1:500 |
| CD8a | BioLegend | 100740 | 1:100 |
